# Patient specific methods for room‐mounted x‐ray imagers for monoscopic/stereoscopic prostate motion monitoring

**DOI:** 10.1002/acm2.12092

**Published:** 2017-05-04

**Authors:** M. Tynan R. Stevens, Dave D. Parsons, James L. Robar

**Affiliations:** ^1^ Department of Radiation Oncology Dalhousie University Halifax NS Canada; ^2^ Department of Physics and Atmospheric Science Dalhousie University Halifax NS Canada

**Keywords:** intrafraction motion, monoscopic, patient specific, prostate cancer, stereoscopic, x‐ray imaging

## Abstract

**Purpose:**

To investigate the improvement of combined monoscopic/stereoscopic prostate motion monitoring with room‐mounted dual x‐ray systems by adopting patient specific methods.

**Methods:**

The linac couch was used as a motion stage to simulate 40 highly dynamic real patient prostate trajectories. For each trajectory, 40 s pretreatment and 120 s treatment periods were extracted to represent a typical treatment fraction. Motion was monitored via continuous stereoscopic x‐ray imaging of a single gold fiducial and images were retrospectively divided into periods of stereoscopic and monoscopic imaging to simulate periodic blocking of the room‐mounted system by the gantry during arc‐based therapy. The accuracy of the combined motion monitoring was assessed by comparison with the linac couch log files. To estimate 3‐D marker position during monoscopic imaging, the use of population statistics was compared to both maximum likelihood estimation and stereoscopic localization based estimation of individualized prostate probability density functions (PDFs) from the pretreatment period. The inclusion of intrafraction updating was compared to pretreatment initialization alone.

**Results:**

Combined mono/stereoscopic localization was successfully implemented. During the transitions from stereoscopic to monoscopic imaging, fiducial localization exhibits sharp discontinuities when population PDFs were employed. Patient specific PDFs successfully reduced the localization error when estimated from stereoscopic localizations, whereas maximum likelihood estimation (MLE) was too unstable in the room‐mounted geometry. Intrafraction stereoscopic updating provided further increases in accuracy. Residual error tended to decrease throughout the treatment fraction, as the patient‐specific PDFs became more refined.

**Conclusions:**

This is the first demonstration of toggled monoscopic/stereoscopic localization using room‐mounted dual x‐ray imagers, enabling continuous intrafraction motion monitoring for these systems. We showed that both pretreatment individualization and intrafraction updating should be used to provide the most accurate motion monitoring.

## 1 Introduction

**Figure 1:**
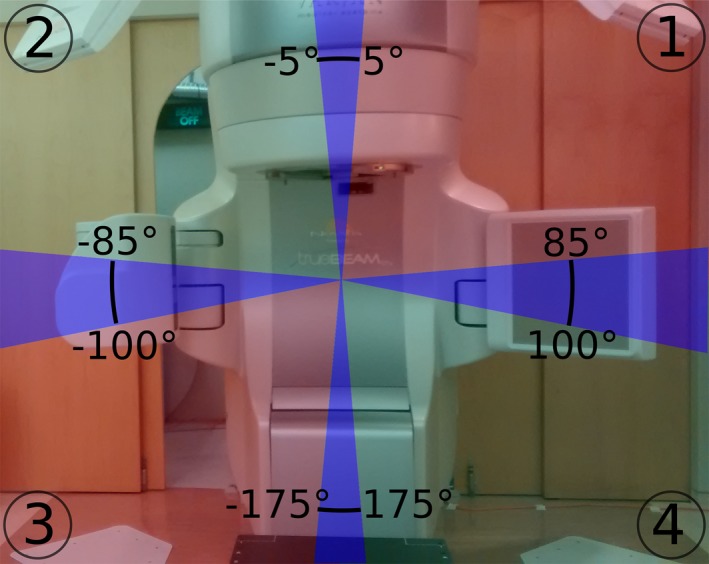
Typical room‐mounted dual x‐ray imaging geometry. The x‐ray detectors (1 and 2) and sources (3 and 4) can be seen in the upper and lower corners of the image, respectively. Stereoscopic imaging is available only for small ranges of angles about each cardinal position (blue shaded areas). For the remaining gantry angles only monoscopic imaging is possible, using either the tube on the right side (red shaded regions) or left side (green shaded regions) of the room.

In modern image guided stereotactic radiation therapy for the prostate, the position of the target may be confirmed at the start of each treatment fraction using a combination of planar imaging and cone‐beam CT. However, intrafraction prostate motion of 1 cm or more is not uncommon,^1^, ^2^ requiring relatively large margins to ensure adequate target coverage,^3^ especially in hypofractionated settings.^4^, ^5^, ^6^ Alternatively, intrafraction monitoring can be used to gate treatment or track prostate motion, in order to ensure accurate dose delivery, for instance using intrafraction x‐ray imaging^7^, ^8^, ^9^, ^10^, ^11^, ^12^, ^13^ or electromagnetic transponders.^1^, ^2^, ^3^, ^14^, ^15^ Continuous monitoring during treatment is possible using room‐mounted stereoscopic x‐ray systems, but the treatment head periodically blocks the x‐ray sources or detectors, for example, as it rotates during a volumetric modulated arc therapy (VMAT) treatment.^16^ Although quad‐x‐ray systems have been demonstrated to overcome this limitation,^7^ the geometry of a typical room‐mounted x‐ray system is such that stereoscopic imaging is only available for approximately 50–60° of the 360° gantry rotation (see Fig. 1).

While exact 3‐D localization is not possible from a single (i.e., monoscopic) planar image, 3‐D position estimation during the monoscopic imaging periods is possible by exploiting the correlated prostate motion typical exhibited in the anterior/posterior (AP) and superior/inferior (SI) directions.^10^ In this approach, the variance/covariance matrix is used to generate a probability density function (PDF) for the prostate position. The x‐ray source point and imaged location of an object (e.g., a fiducial marker) define a 1D ray line through this PDF, from which the most likely fiducial position can be estimated as the mean position of the 1D‐PDF along that ray line.

In the original implementation of this method,^10^ the monoscopic images were obtained from the rotating on‐board‐imager (OBI) of the Linac, and the PDF was produced from population statistics from a large Calypso‐based study.^1^ However, in subsequent studies, Poulsen et al.^11^ showed that patient‐specific PDFs were more accurate than population statistics for monoscopic localization. In order to generate individualized PDFs from OBI image data, Poulsen et al. used a maximum likelihood estimation (MLE) technique, in which the PDF parameters that maximized the probability of all observed image locations were determined. In their study, two individualization schemes were investigated: using a pretreatment period to generate the patient‐specific PDF (“static”), and continuing to update the PDF parameters during treatment (“dynamic”). The best results were obtained from the dynamically updated PDFs.

We previously studied the accuracy of monoscopic localization using a room‐mounted x‐ray imaging system and population variance/covariance statistics.^16^ It is natural to anticipate that individualized PDF parameters will also improve the accuracy of monoscopic localization for room‐mounted geometry. However, it is not obvious that the MLE based approach is ideal for room‐mounted imaging geometry, as the monoscopic projections obtained are all closely aligned to the two principal imaging directions, and thus the amount of independent information available for maximum likelihood estimation is low compared to the rotating imager case. We will show that for room‐mounted geometry, direct measurement of the PDF parameters from the intermittent stereoscopic imaging windows can be used instead of MLE estimation to improve the accuracy of monoscopic localization during treatment. This novel methodological approach was not possible in previous implementations of monoscopic motion monitoring, due to the availability of only a single kV imaging panel.

In this study we further demonstrate that methodological improvements can reduce the residual monoscopic localization error when using a room‐mounted x‐ray system. In our recent study,^16^ we showed that the residual localization error during monoscopic imaging depended on the particular trajectory, and which monoscopic view was available. In general, the largest residual error occurred during the largest excursions from baseline, and we therefore investigated a series of particularly challenging prostate trajectories with large amounts of intrafraction motion, as these have the highest potential to show significant advantages of one method over another. Furthermore, in this study we use a realistic implementation of room‐mounted tracking, in which the localization toggles between monoscopic and stereoscopic monitoring depending on gantry angle. Our main hypothesis is that location based PDF estimation will outperform MLE based estimation for the room‐mounted geometry. We will also investigate the effects of dynamic updating of PDFs during treatment and the use of three different imaging rates (1, 2, and 4 Hz) for motion monitoring.

## 2 Methods

### 2.A Prostate trajectories

Forty prostate trajectories were extracted from a database of 550 patient datasets (2.5–18.4 min duration) obtained from a previous study.^1^ The entire database was first sorted in terms of total variance in position. The 40 most dynamic trajectories were selected, as the largest errors are expected during large excursions, and therefore methodological improvements should have the greatest impact in these cases. For each of the selected trajectories, a 40 s period was extracted from the start of the trajectory, to represent a pretreatment period during which prostate motion could theoretically be monitored stereoscopically. A 120 s treatment period was also extracted, using a time window selected to contain the largest possible amount of motion. In the case where this treatment window was at the start of the trajectory, the pretreatment window was moved to after the selected treatment period.

Each trajectory was converted to an XML file, which used to control the treatment couch using developer mode on the Linac (Varian STx, Varian Medical Systems, Inc.), as described previously.^16^ A single cylindrical gold fiducial was placed directly on the couch at isocentre. In order to eliminate the effects of setup error, the fiducial was initially imaged in a static position to calibrate the isocentre position on the x‐ray images (i.e., all motion was assessed relative to this baseline position).

### 2.B Imaging Technique

Imaging was performed using a dual room‐mounted x‐ray system (Exactrac, Brainlab AG, Feldkirchen, Germany). Continuous stereoscopic imaging was performed at 4 Hz during both the pretreatment and treatment trajectories, using a technique of 140 kVp and 1.0 mAs. Images were transferred from the acquisition and analyzed retrospectively.

### 2.C Combined stereo/monoscopic localization

During a realistic treatment fraction, the x‐ray tubes would be periodically blocked by the rotating gantry as described above. Therefore, in order to simulate a realistic implementation of continuous intrafraction monitoring, images were retrospectively divided into stereoscopic and monoscopic periods based on the gantry angle. A continuous gantry rotation at constant speed was assumed in order to assign images to the appropriate stereoscopic or monoscopic segment. The resulting trajectories contain five distinct stereoscopic periods of four to twenty images, separated by four periods of approximately 100 monoscopic images (from the left or right x‐ray source depending on which quadrant the treatment head was in). An example of this combined monoscopic/stereoscopic localization is shown below.

#### 2.C.1 PDF parameter estimation

As a baseline comparison for the individualized PDF parameter estimation methods, we used the same population covariance matrix (“**C**”) as in previous publications,^10^, ^16^ which was derived from the same patient database used in this study:

$$C=\left[\begin{array}{ccc} {\mathrm{var}}_{x} & {\mathrm{cov}}_{xy} & {\mathrm{cov}}_{xz}\\ {\mathrm{cov}}_{xy} & {\mathrm{var}}_{y} & {\mathrm{cov}}_{yz}\\ {\mathrm{cov}}_{xz} & {\mathrm{cov}}_{yz} & {\mathrm{var}}_{z} \end{array}\right]=\left[\begin{array}{ccc} 0.3163 & -0.0775 & 0.0114\\ -0.0775 & 2.4733 & 1.5051\\ 0.0114 & 1.5051 & 1.8820 \end{array}\right]mm^{2}$$

Where the x, y, and z directions correspond to patient left/right, superior/inferior and anterior/posterior, respectively. Three PDF parameter initialization schemes were investigated: none (i.e., population statistics only), location based pretreatment individualization, and MLE based pretreatment individualized (see below). For each PDF estimation scheme, the mean and maximum error (3‐D distance between localizations obtained from the images and the reported location from the linac log file at that time) over the course of each extracted treatment fraction was calculated. Finally, three imaging rates were assessed for intrafraction updating (1, 2 ,and 4 Hz).

#### 2.C.2 Initialization strategies

**Figure 2:**
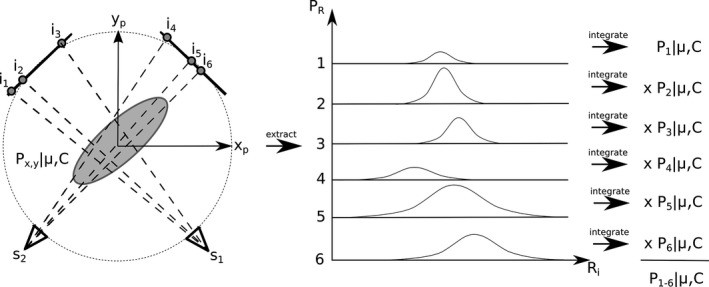
Maximum likelihood estimation of the PDF parameters (shown in 2D for simplicity). The 2D guassian PDF (represented by the gray shaded region) is parameterized by μ (the mean position) and C (the covariance matrix). Maximum likelihood estimation of these parameters consists of extraction of 1D PDFs along each ray line defined by each of the “N” detected image points (i_i_), and corresponding source locations (s_1_ or s_2_ depending on the detector the image is from). Integration of these 1D PDFs gives the probability of observing the fiducial at that location in the image given a set of PDF parameters (P_i_|μ,C). The total probability of all observed image locations (P_1‐N_|μ,C) is calculated as the product of the individual image probabilities, and the MLE PDF parameters are those that maximize this total probability.

MLE based PDF initialization was performed in the same fashion as described in Poulsen et al.^11^ In this approach, observed image locations from the pretreatment period of an individual patient are used to estimate a patient‐specific PDF. The image locations are assumed to be sampled from a 3‐D guassian distribution defined by variance/covariance values and mean positions relative to isocentre in x, y, and z directions. The probability of observing a fiducial at a given image location is equal to the integral of the 1‐D guassian distribution sampled from the full 3‐D distribution along the ray line connecting the source/image locations (see Fig. 2). The total probability of all observed image locations is then given by the product of the individual image probabilities, and depends on the parameters of the 3‐D gaussian. By maximizing the total probability of all observed image locations, the most likely PDF parameter set can be estimated. In the current implementation, this amounts to two separate lists of image locations as input (one for each x‐ray panel). The most robust results were obtained by iterating the MLE solution as image pairs were added, using the outputs (i.e., estimated PDF parameters) of each iteration as the initial values for the subsequent iteration.

Conversely, location based initialization of the PDF parameters involves the direct calculation of the observed variance/covariance matrix and mean position from the pretreatment window localizations. For this purpose, a list of observed fiducial locations in 3‐D space is created by stereoscopic reconstruction of the fiducial location in each image pair as detailed in Stevens et al.^16^ The location based approach requires no modeling and makes no assumption about the statistical distribution of the pretreatment motion trajectory. This approach is uniquely viable using stereoscopic imaging systems as it relies on knowing the 3‐D location of the fiducial during pretreatment, and therefore was not possible in the previously studied implementations of monoscopic motion monitoring that used the OBI system.

#### 2.C.3 Intrafraction Updating

The PDF parameters calculated during pretreatment can be updated during the treatment period using the images already being collected for continuous fiducial monitoring. This will likely be most important when the motion observed during pretreatment is not representative of the motion that occurs during treatment, that is, a scenario that cannot be predicted in advance. For the MLE based method, updating is accomplished by appending new observed image locations to the lists of pretreatment image locations, and recalculating the most likely PDF parameters. For the location based method, each new localization is appended to the list of observed positions, and the variance, covariance, and mean position are recalculated.

#### 2.C.5 Image Rate Effects

The effect of imaging rate was assessed only for the best combination of PDF initialization and intrafraction updating identified above. For this purpose, the 4 Hz data obtained experimentally was decimated to 2 or 1 Hz, and the localization procedure (including PDF estimation) was repeated. The mean error calculation was implemented differently for the image frequency assessment, because image rate not only affects accuracy at the time of imaging, but also the lag between the prostate trajectory and localizations. Therefore, for the imaging frequency assessment, we defined instead the mean integrated error as follows: the imaging time‐points were interpolated onto the high temporal resolution of the linac log file (50 Hz), and each imaged location was assumed to apply from the first time‐point after the image was obtained until the last time‐point before the next image was taken. The error versus time can then be calculated as the difference in x/y/z location of these (temporally) interpolated image locations to the locations in the linac log, and the mean integrated error can be computed by integrating the error magnitude and dividing by the total time (i.e., 120 s for the treatment period).

#### 2.C.6 Residual Error Characterization

The residual motion statistics for the population PDF and the best individualized PDFs identified above were compared to the idealized case of full continuous stereoscopic imaging, in order to separate the error due to the monoscopic algorithm accuracy from that of the imaging system as a whole. Furthermore, for each time point in each trajectory examined, the residual error of the best method for individualized PDF estimation was compared to the displacement from baseline at that time. From this analysis, the frequency of displacements and residual errors was determined and plotted as a 2‐D color‐map. Finally, we examined the residual error as a function of time during treatment, averaged over each quadrant, in order to show how the continual updating of PDF parameters continues to improve the motion monitoring technique as treatment progresses. This is especially important in the context of hypofractionated treatment regimens, which may involve longer treatment fraction durations.

## 3 Results

### 3.A Combined stereo/monoscopic localization

**Figure 3:**
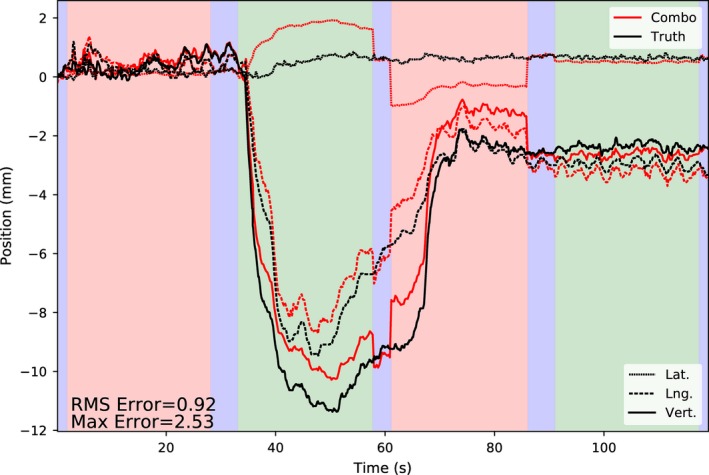
Example motion monitoring result using the combined stereoscopic/monoscopic localization technique (i.e., “combo” imaging). The blue/green/red shaded regions correspond to the gantry angles shown in Fig. 1, and therefore to where stereoscopic or monoscopic imaging are available. Localization discontinuities are observed when switching from monoscopic to stereoscopic localization, and estimation errors during large excursions due to deficiencies with the population PDF for predicting this trajectory are also seen. The ground truth data was taken from the linac log files.

An example of the combined stereoscopic and monoscopic localization during a representative treatment arc using the population variance/covariance matrix is shown in Fig. 3. During the first large excursion around t = 35 s, the population covariance overestimates the amount of motion in the left/right direction, and underestimates the motion in the other two dimensions. Around t = 60 s, stereoscopic imaging becomes available again, and the localization becomes very accurate, at the expense of a discontinuity in the estimated position. During the next monoscopic period, the other imaging panel is unblocked, and the left/right error is biased in the opposite direction. These discontinuities, left/right bias error, and underestimation of motion in the sup/inf and ant/post directions are characteristic of the combined stereoscopic/monoscopic localization using population averaged PDF parameters in highly dynamic prostates.

### 3.B Pretreatment PDF individualization

**Figure 4:**
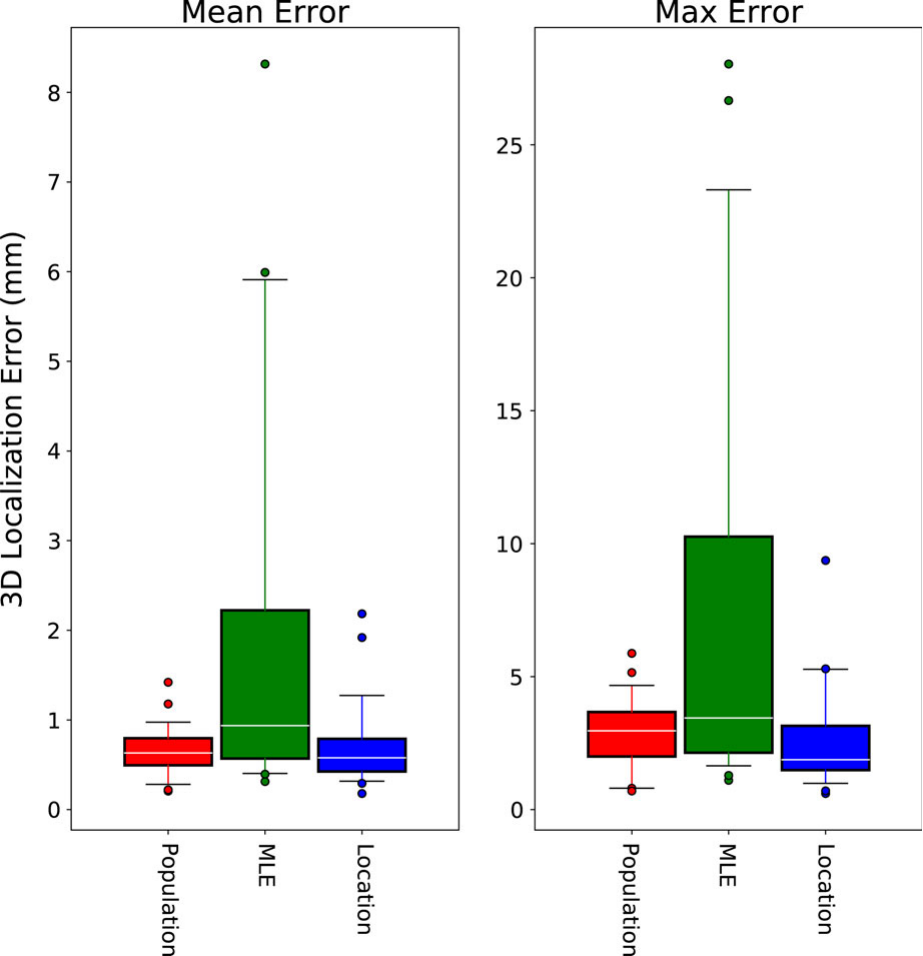
Mean and maximum intrafraction localization error for the three PDF Initialization schemes assessed: none (population statistics only), MLE‐based individualized, and location‐based individualization. The boxes show the 25^th^ to 75^th^ percentiles, with the median represented by white lines. The whiskers indicate the 5^th^ to 95^th^ percentiles, and outliers are shown as circles.

Pretreatment individualization of the PDF parameters was significantly more accurate using the stereoscopic location based approach than MLE. Although MLE sometimes produced suitable PDF parameters for motion monitoring, there were a number of cases in which large errors (i.e., >5 mm) were incurred, as evidenced by the large range between the median and 75^th^ percentile for the mean and maximum errors using MLE (Fig. 4). In a few extreme outlier cases, MLE individualization resulted in more residual error than no tracking at all.

**Figure 5:**
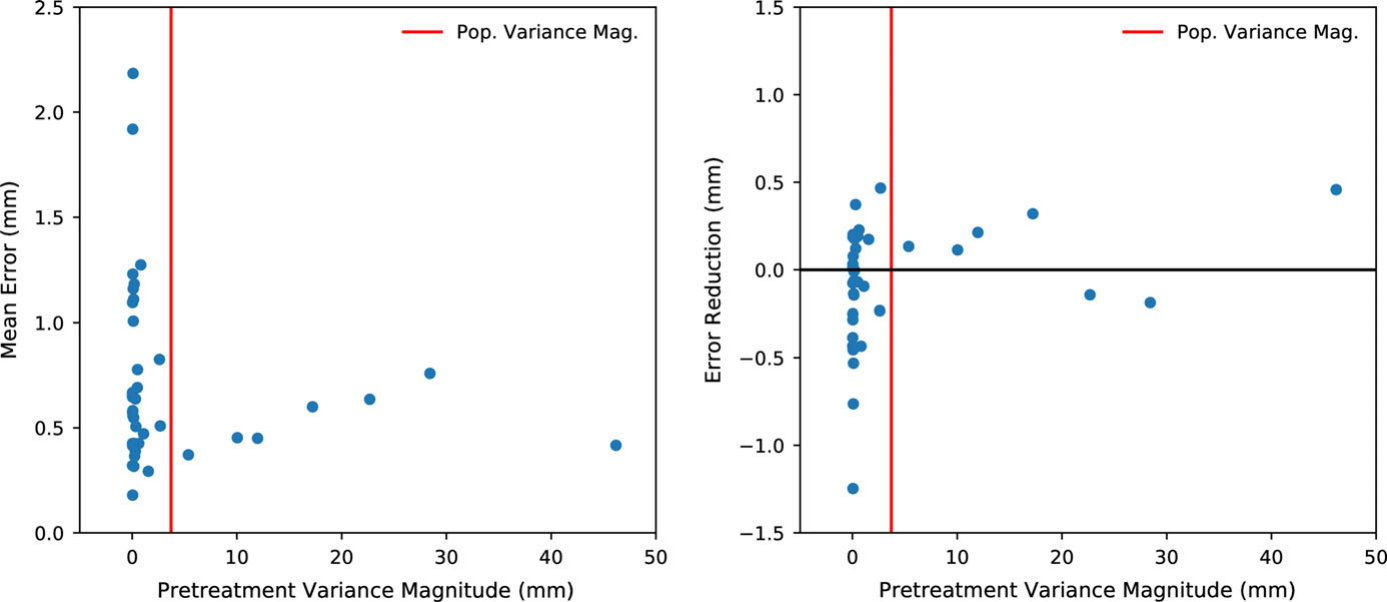
The residual localization error for location‐based PDF individualization versus the amount of variance in the pretreatment period (left), and the reduction in residual error compared to using the population PDF (right). For reference, the magnitude of the average population variance is shown by the red line. The prostate trajectories with larger (>1.0 mm) residual error had very little motion during the pretreatment period, resulting in PDFs that were not predictive of the motion observed during treatment. For many of these trajectories, the population PDF produced more accurate localization.

By contrast, location based PDF initialization was in most cases better than using population statistics, resulting in reductions of the median and 25^th^ percentiles of the mean error (0.63–0.58 mm and 0.49–0.43 mm, respectively) as well as the median, 25^th^ and 75^th^ percentiles of the maximum error (2.96–1.88 mm, 2.00–1.49 mm and 3.67–3.15 mm, respectively). However, there were a few outlier datasets for which the individualized PDFs produced less accurate motion monitoring results than the population PDF. In most of these cases, it was noted that there was very little motion in the extracted pretreatment trajectory. Figure 5 illustrates this, by comparing the mean residual error using individualized PDF to the amount of pretreatment variance.

### 3.C Intrafraction PDF updating

**Figure 6:**
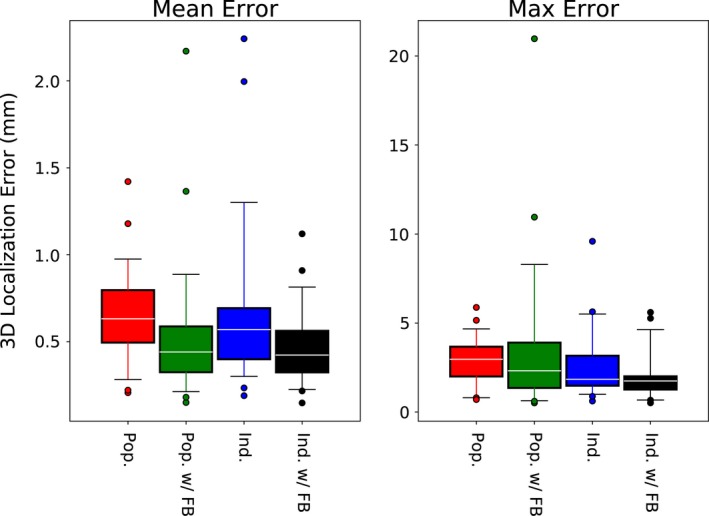
Mean and maximum intrafraction localization error with (w/FB) and without intrafraction stereoscopic feedback, and with (Ind.) and without (Pop.) pretreatment initialization. In general the residual errors were reduced by the inclusion of intrafraction feedback, especially when combined with pretreatment initialization. In the case of no pretreatment individualization with intrafraction updating, the initial PDF used for monoscopic localization was formed from just the few images acquired in the first 5° of the treatment arc. This resulted in larger maximum errors, as the variance/covariance estimates during the first monoscopic period were based off an insufficient amount of data.

Mean error was reduced substantially by updating the PDF parameters during treatment when stereoscopic localization was available, whether or not the pretreatment period was used to initialize the PDFs (Fig. 6). In the latter case, the PDF available during the first monoscopic period was formed from only the relatively few (~8–10) stereoscopic images available at the start of the treatment arc. Because of this, there were occasionally large errors during this first monoscopic quadrant, and thus the maximum error of localization using intrafraction updating without pretreatment initialization had some extreme outliers. The pretreatment individualized PDFs with intrafraction updating were especially effective at reducing the maximum localization error compared to other methods.

### 3.D Error vs imaging rate

**Figure 7:**
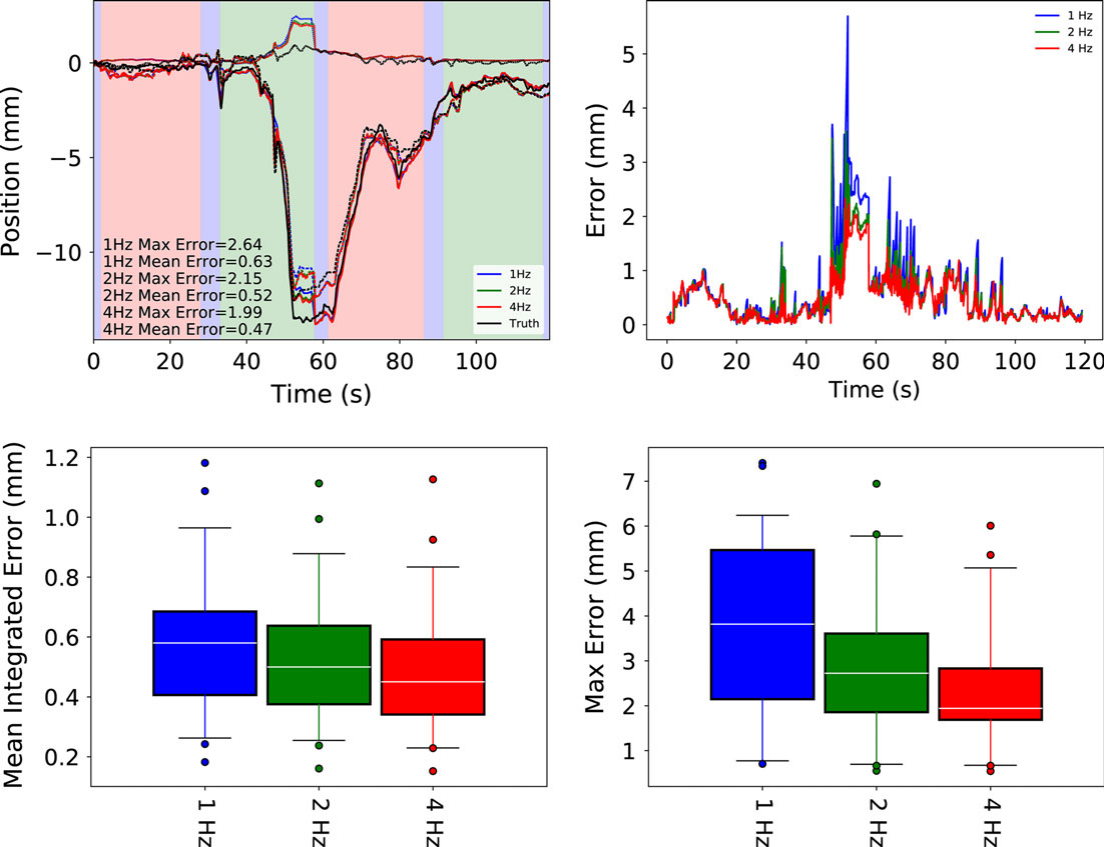
Example trajectory monitored at 1, 2, and 4 Hz (top left), the residual error magnitude over time for that trajectory (top right), the mean integrated error for all 40 trajectories investigated (bottom left), and the corresponding maximum intrafraction localization error (bottom right). Most of the increased error at low imaging rates occur briefly during large excursions, although around t = 55 s a period of elevated error for 1 Hz imaging can be observed.

The mean integrated error and the maximum intrafraction localization error both decrease as the imaging rate is increased from 1 to 4 Hz (median across individual trajectories from 0.58 to 0.45 mm and 3.81 to 1.94 mm, respectively). Sustained elevated errors at low imaging rates tended to occur during the first large excursion, whereas brief spikes in residual error occur throughout the treatment period during rapid movements as the image data lags further behind the true trajectory (Fig. 7).

#### 3.D.1 Residual Error Characterization

**Figure 8:**
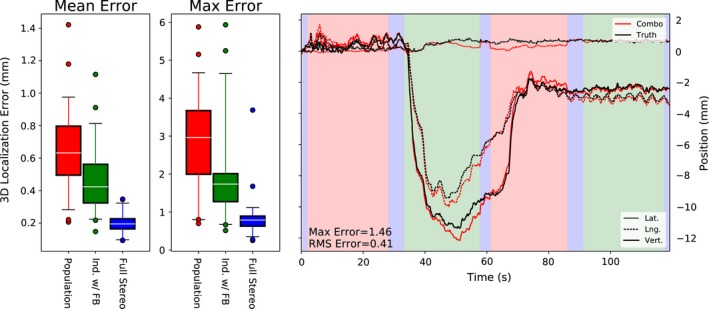
Localization accuracy of the 4 Hz combined mono/stereoscopic imaging using population statistics of individualized PDFs compared to the practical limit of full stereoscopic monitoring (left), and the same example trajectory as shown in Fig. 3, now monitored using the best individualized method (right).

In Fig. 8, the residual error is compared for three scenarios: motion monitoring with population statistics only, with pretreatment individualized and intrafraction updating, and in the theoretical limit of full stereoscopic imaging throughout the trajectory. The full stereoscopic case demonstrates the portion of the residual error due to system characteristics including detector resolution and fiducial detection accuracy. These factors should also be present in the monoscopic methods, providing an upper limit on monitoring accuracy. Both in terms of mean and maximum intrafraction localization error, about half of the potential improvement in accuracy is realized (e.g., mean error reduced from 0.65 ± 0.04 mm to 0.47 ± 0.03 mm compared to the stereo limit of 0.20 ± 0.01 mm). Also shown in Fig. 8 is the same trajectory illustrated in Fig. 3, now monitored using the best patient‐specific method. In this case, the discontinuities when switching from stereoscopic to monoscopic imaging are nearly resolved completely.

#### 3.D.2 Error vs displacement

**Figure 9:**
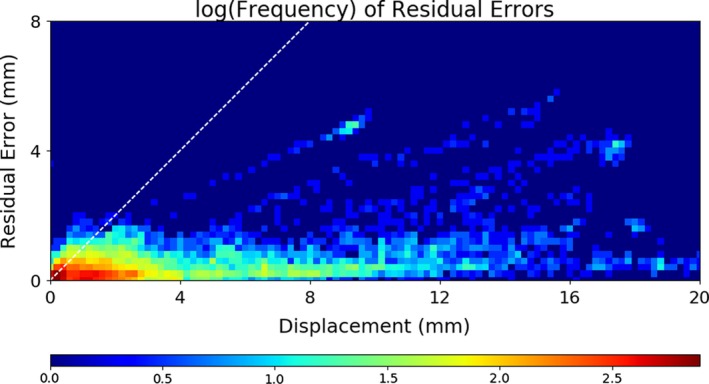
Natural log frequency of the residual errors plotted as a function of displacement for all time points of all trajectories investigated. The majority of time points have both low displacement (<4 mm) and low residual error (<2 mm). For reference, the white dashed line shows equal error to displacement, as would be the case if no monitoring were employed.

The residual errors for every time point in each trajectory are summarized in Fig. 9. No large errors were observed for small displacements, showing that the individualized methods do not produce significant false motion estimates due to the shifting of the mean PDF position. Even for highly dynamic prostate trajectories, the majority of time points have relatively low displacements (<4 mm) and residual error (<2 mm). Even for relatively large excursions of 1 cm or more, the majority of the residual errors are less than 2 mm, although traces of individual excursions with larger than typical residual errors can also be observed.

#### 3.D.3 Error over time

**Figure 10:**
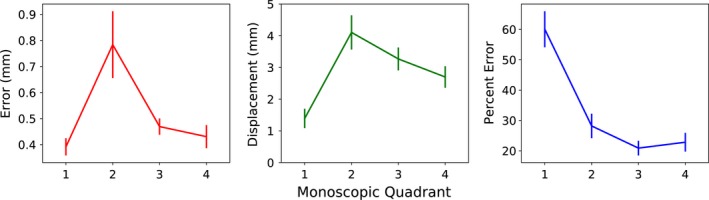
Residual error (left) and trajectory displacement (middle) as a function of time during treatment, averaged over the four monoscopic quadrants. The residual error and displacement increase rapidly from the first to second quadrant, but the error then decreases in later quadrants, whereas the displacement remains relatively elevated. The percent residual error steady decreases throughout the treatment fraction (right).

The residual error was averaged for each of the four quadrants of gantry rotation. During the first quadrant, both the localization error and displacement from baseline were low (0.39 ± 0.03 mm and 1.39 ± 0.30 mm, respectively), as the initial setup was recently performed (Fig. 10). Both displacement and error spike during the second quadrant (0.78 ± 0.13 mm and 4.11 ± 0.54 mm, respectively), as many of the large excursions have begun to occur at this point. However, while the displacement remains elevated from this point onward (due to both later occurring excursions and slow drift type motions), the monitoring error decreases as the individualized PDFs become more and more accurate due to intrafraction updating. When expressed as a percentage of displacement, the residual error steadily decreases throughout the trajectory.

## 4 Discussion

We investigated combined monoscopic/stereoscopic intrafraction motion monitoring for fiducial localization during prostate treatments using a dual room‐mounted x‐ray system. This is the first demonstration of toggling between monoscopic and stereoscopic localization as the gantry rotates and blocks one or the other x‐ray imager. The seamless transition between imaging modes allows for continuous intrafraction monitoring, and can be combined with treatment gating, couch or MLC tracking for more accurate delivery of treatment.^17^, ^18^, ^19^, ^20^, ^21^ This is especially important for hypofractionated treatment regimens, where geometric miss during a single fraction is more impactful on overall dose distribution due to the low number of treatment fractions.

We also demonstrated that individualized PDFs can be used to produce more accurate localization during the monoscopic periods, reducing the discontinuities observed during stereo/mono transitions using population averaged PDFs. This was especially apparent in the sub‐sample of highly mobile prostate trajectories examined in this study, as these trajectories are not well represented by the overall population average. As there is no way to anticipate beforehand which patients will resemble the population motion covariances, and it is these highly dynamic prostates that are most important to monitor accurately, the reduction in residual error provided by PDF individualization is vital. The reduction of maximum error from 2.8 ± 0.2 mm to 2.0 ± 0.2 mm we observed when using patient‐specific methods is especially important in terms of reducing the potential for geometric miss in dose delivery.

Compared to previous studies on PDF individualization using an OBI system,^11^ the mean error in our study was higher (0.47 ± 0.03 mm compared to 0.22 mm), whereas the maximum error was significantly lower (2.0 ± 0.2 mm compared to 4.5 mm). This is likely because the sample of prostate trajectories examined in this study was a subset of those reported in Poulsen et al., and specifically the most dynamic cases. These trajectories are thus much further from their baseline position on average, and therefore larger absolute errors can be expected. The fact that the maximum error in our results was lower than the previous study suggests that the stereoscopic PDF estimation is more efficient than the MLE approach at responding to large excursions.

One potential limitation of our study is that we restricted our investigation to large amplitude prostate motions. In the average prostate cancer patient, smaller prostate excursions are expected, and intrafraction motion monitoring may add unnecessary complexity to treatment execution. Yet even for patients with stable pretreatment prostate trajectories, prostate dynamics during treatment are unpredictable. Unanticipated motion (e.g., persistent excursion due to rectal filling) could be detrimental to dosimetric coverage. Any motion monitoring scheme employed should thus be able to deal with these extreme cases. A second possible criticism is that the data used to build the population PDFs employed in this study included the subset of motion trajectories under investigation, which (a) would not be available in the clinical setting and (b) could bias the results towards the use of population averages. However, the best method identified in this study used only individualized pretreatment and intrafraction PDF estimation, minimizing the relevance of this shortcoming.

In previous studies using the kV on‐board imager for monoscopic tracking, it was shown that a maximum likelihood estimation technique was able to produce individualized pretreatment PDFs that improved monitoring accuracy.^11^ However, in our study we found that the MLE method was highly unstable for room‐mounted geometry. The reason for this discrepancy is likely that the rotating frame of reference of the OBI provides many independent orientations to sample the PDF whereas for the room‐mounted geometry the projection angles are essentially fixed, and so all fiducial projections are roughly aligned to these two principal axes. This results in a set of ray‐line samples that could be explained by many different PDFs, and thus a poorly specified minimization problem. The direct measurement of the PDF parameters from stereoscopic localization during pretreatment was more accurate for room‐mounted geometry, and when updated throughout the treatment during the stereoscopic windows produced the most accurate localization results. It is worth emphasizing that this direct PDF measurement approach is not possible with a single kV imager system employed in previous studies using the OBI, and therefore represents a major advantage to our novel approach.

We observed a greater impact of imaging frequency on localization accuracy than previous studies,^11^ which we hypothesize is due to the relatively brief periods during treatment for which stereoscopic imaging is available, resulting in very little information begin added to the PDF estimates at low imaging rates. Most of the reduced accuracy was exhibited as brief residual error spikes as the monitoring lagged behind the motion during rapid transitions. In general the dosimetric impact of this would likely be small, even in hypofractionated settings, suggesting that imaging dose (previously estimated at about 15.3 μGy per image in the vicinity of the rectum^16^) could be reduced by using lower imaging rates. More problematic is the occasional observation that early in the trajectory the lower imaging rate produced prolonged elevated error, again presumably because fewer image samples were available to form a good estimate of the PDF parameters. Increasing the imaging rate above 4 Hz could potentially increase localization accuracy even further, however, at some point an acceptable amount of unresolved motion must be defined. A balance between lowering image rate (and therefore kV dose) and monitoring accuracy could potentially be achieved either by having higher imaging rates during the stereoscopic windows than during monoscopic imaging, or by having the imaging rate start high, and decrease over the course of treatment.

The trends in localization error as a function of time during treatment were interesting for multiple reasons. Firstly, it has been shown previously that over large populations, the prostate position tends to drift further and further from baseline over prolonged treatment times.^1^, ^22^ This was somewhat consistent with our results, although it should be noted that a predominance of transient excursions over low frequency drifts in the selection of high variance trajectories somewhat reduced the displacements at later times. We observed that the localization error actually tends to decrease later in the treatment fraction when intrafraction updating is employed. In fact, when viewed as a percentage of displacement, localization error decreases continuously throughout treatment. Thus as the treatment fraction continues, the typically larger and larger prostate displacements will have less and less detrimental effect on the dose delivery using the proposed monitoring method. This is especially important for hypofractionated treatment regimens, which may have extended fraction durations. There may also be some potential to adjust the monitor‐unit weighting over a treatment fraction to deliver more dose later in the fraction when the localization uncertainty is lowest, although this idea would require further study.

## 5 Conclusion

We have demonstrated for the first time an implementation of combined stereoscopic/monoscopic localization using a room‐mounted x‐ray system to overcome gantry blocking issues and to enable continuous intrafraction monitoring. Furthermore, we demonstrated significant improvements in localization accuracy when using patient specific methods. Specifically, employing both pretreatment initialization and intrafraction updating from direct stereoscopic measurement of the variance, covariance, and mean position resulted in the most accurate intrafraction monitoring for this imaging system. These improved motion monitoring capabilities can potentially be leveraged to improve the accuracy of treatment delivery.

## Conflict of interest

The authors have no relevant conflicts of interest to disclose.

## Figures and Tables

**Figure 11:**
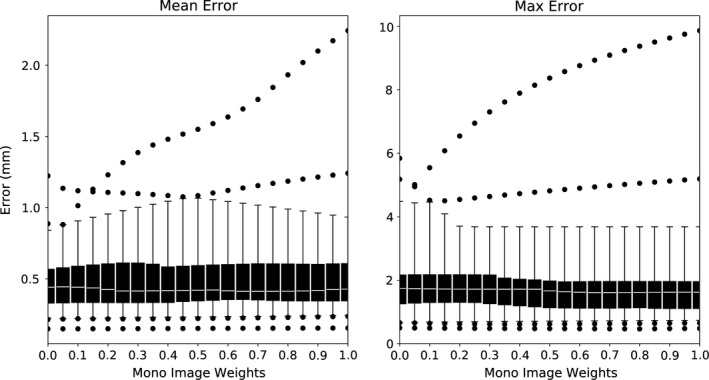
Mean and maximum intrafraction localization error when including localizations from the monoscopic periods in intrafraction updating. The monoscopic weights were used both for calculating weighted average for the mean position and weighted covariances for individualizing the PDFs. While there was a trend towards lower maximum error around monoscopic a weighting of 0.5, inclusion of the monoscopic updating did not provide consistent benefits.

## Appendix 1 – Monoscopic PDF updating 1

The utility of intrafraction stereoscopic PDF updating leads to the question of whether or not localization data from throughout the treatment fraction (i.e., monoscopic as well as stereoscopic) could further improve the response to changes in prostate motion dynamics during the treatment fraction. To test this, we assigned relative weighting of between 0 and 1 to the monoscopic localizations, in order to determine whether equal importance should be placed on the estimated locations (i.e., monoscopic) as the directly observed ones (i.e., stereoscopic).

The inclusion of localizations from the monoscopic image periods to the updating of the PDF parameters had equivocal results (Fig. 11). While there was a trend to reduce the maximum localization error with increasing weight applied to the monoscopic images, this effect saturated above a weighting factor of 0.5. Furthermore, there was no clear effect on the mean intrafraction residual error, and one outlier dataset actually exhibited a substantial increase in both mean (0.9–2.3 mm) and maximum (5.2–9.9 mm) error as the monoscopic feedback weight was increased from 0 to 1. This is likely caused by propagating errors in monoscopic localization back into the PDF parameters, forming a positive feedback loop and amplifying the resulting error. Therefore, the inclusion of monoscopic feedback can thus not be recommended, as the modest increases in average accuracy do not justify the risk of introducing occasional large localization errors.

## References

[1] Langen KM , Willoughby TR , Meeks SL , et al. Observations on real‐time prostate gland motion using electromagnetic tracking. Int J Radiat Oncol. 2008;71:1084–1090. 10.1016/j.ijrobp.2007.11.054 18280057

[2] Su Z , Zhang L , Murphy M , Williamson J . Analysis of prostate patient setup and tracking data: Potential intervention strategies. Int J Radiat Oncol. 2011;81:880–887. 10.1016/j.ijrobp.2010.07.1978 PMC3020989 20934274

[3] Tanyi JA , He T , Summers PA , et al. Assessment of planning target volume margins for intensity‐modulated radiotherapy of the prostate gland: Role of daily inter‐ and intrafraction motion. Int J Radiat Oncol. 2010;78:1579–1585. 10.1016/j.ijrobp.2010.02.001 20472357

[4] Hegemann N‐S , Guckenberger M , Belka C , Ganswindt U , Manapov F , Li M . Hypofractionated radiotherapy for prostate cancer. Radiat Oncol. 2014;9:275. 25480014 10.1186/s13014-014-0275-6 PMC4273481

[5] Li HS , Chetty IJ , Enke CA , et al. Dosimetric consequences of intrafraction prostate motion. Int J Radiat Oncol. 2008;71:801–812. 10.1016/j.ijrobp.2007.10.049 18234439

[6] Adamson J , Wu Q , Yan D . Dosimetric effect of intrafraction motion and residual setup error for hypofractionated prostate intensity‐modulated radiotherapy with online cone beam computed tomography image guidance. Int J Radiat Oncol. 2011;80:453–461. 10.1016/j.ijrobp.2010.02.033 PMC3010484 20646842

[7] Shimizu S , Shirato H , Kitamura K , et al. Use of an implanted marker and real‐time tracking of the marker for the positioning of prostate and bladder cancers. Int J Radiat Oncol Biol Phys. 2000;48:1591–1597. 11121666 10.1016/s0360-3016(00)00809-9

[8] Adamson J , Wu Q . Prostate intrafraction motion evaluation using kV fluoroscopy during treatment delivery: A feasibility and accuracy study. Med Phys. 2008;35:1793. 18561654 10.1118/1.2899998 PMC2507873

[9] Adamson J , Wu Q . Prostate intrafraction motion assessed by simultaneous kV fluoroscopy at MV delivery II: Adaptive strategies. Int J Radiat Oncol. 2010;78:1323–1330. 10.1016/j.ijrobp.2009.09.079 PMC2948079 20584578

[10] Poulsen PR , Cho B , Langen K , Kupelian P , Keall PJ . Three‐dimensional prostate position estimation with a single x‐ray imager utilizing the spatial probability density. Phys Med Biol. 2008;53:4331–4353. 18660559 10.1088/0031-9155/53/16/008

[11] Poulsen PR , Cho B , Keall PJ . Real‐time prostate trajectory estimation with a single imager in arc radiotherapy: A simulation study. Phys Med Biol. 2009;54:4019–4035. 19502704 10.1088/0031-9155/54/13/005

[12] Wiersma RD , Mao W , Xing L . Combined kV and MV imaging for real‐time tracking of implanted fiducial markers. Med Phys. 2008;35:1191. 18491510 10.1118/1.2842072 PMC2811551

[13] Dzierma Y , Nuesken FG , Licht NP , Ruebe C . Dosimetric properties and commissioning of cone‐beam CT image beam line with a carbon target. Strahlenther Onkol. 2013;189:566–572. 23715886 10.1007/s00066-013-0330-5

[14] Willoughby TR , Kupelian PA , Pouliot J , et al. Target localization and real‐time tracking using the Calypso 4D localization system in patients with localized prostate cancer. Int J Radiat Oncol. 2006;65:528–534. 10.1016/j.ijrobp.2006.01.050 16690435

[15] Shah AP , Kupelian PA , Willoughby TR , Langen KM , Meeks SL . An evaluation of intrafraction motion of the prostate in the prone and supine positions using electromagnetic tracking. Radiother Oncol. 2011;99:37–43. 21458092 10.1016/j.radonc.2011.02.012

[16] Stevens MTR , Parsons DD , Robar JL . Continuous monitoring of prostate position using stereoscopic and monoscopic kV image guidance. Med Phys. 2016;43:2558–2568. 27147366 10.1118/1.4947295

[17] Souza WDD’ , Naqvi SA , Yu CX . Real‐time intra‐fraction‐motion tracking using the treatment couch: A feasibility study. Phys Med Biol. 2005;50:4021–4033. 16177527 10.1088/0031-9155/50/17/007

[18] Qiu P , Souza WDD’ , McAvoy TJ , Ray Liu KJ . Inferential modeling and predictive feedback control in real‐time motion compensation using the treatment couch during radiotherapy. Phys Med Biol. 2007;52:5831–5854. 17881803 10.1088/0031-9155/52/19/007

[19] Sawant A , Smith RL , Venkat RB , et al. Toward submillimeter accuracy in the management of intrafraction motion: The integration of real‐time internal position monitoring and multileaf collimator target tracking. Int J Radiat Oncol. 2009;74:575–582. 10.1016/j.ijrobp.2008.12.057 19327907

[20] Poulsen PR , Fledelius W , Cho B , Keall P . Image‐Based Dynamic Multileaf Collimator Tracking of Moving Targets During Intensity‐Modulated Arc Therapy. Int J Radiat Oncol. 2012;83:e265–e271. 10.1016/j.ijrobp.2011.12.053 PMC3351539 22401924

[21] Keall PJ , Cattell H , Pokhrel D , et al. Geometric accuracy of a real‐time target tracking system with dynamic multileaf collimator tracking system. Int J Radiat Oncol. 2006;65:1579–1584. 10.1016/j.ijrobp.2006.04.038 16863935

[22] Kotte ANTJ , Hofman P , Lagendijk JJW , Van Vulpen M , Van der Heide UA . Intrafraction motion of the prostate during external‐beam radiation therapy: Analysis of 427 patients with implanted fiducial markers. Int J Radiat Oncol. 2007;69:419–425. 10.1016/j.ijrobp.2007.03.029 17513059

